# Paediatric meningiomas: a multi-centre case series of 27 patients

**DOI:** 10.1007/s00381-024-06684-2

**Published:** 2024-11-29

**Authors:** Luke Rhys Mattey, Zoë James, Taha Lilo, Yazan El Adwan, Maria Rosaria Scala, Ian Kamaly-Asl, Conor Mallucci, Paul Leach

**Affiliations:** 1 Cardiff University School of Medicine, Cardiff, Wales UK; 2 Department of Neurosurgery, University Hospital of Wales, Cardiff, Wales UK; 3 Department of Neurosurgery, Royal Preston Hospital, Preston, UK; 4 Department of Neurosurgery, Royal Manchester Children’s Hospital, Manchester, UK; 5 Department of Neurosurgery, Alder Hey Children’s Hospital, Liverpool, UK

**Keywords:** Case series, Meningioma, Multi-centre

## Abstract

**Purpose:**

This study presents a series of paediatric meningiomas and discusses aetiology, risk factors and outcomes with comparison to current literature.

**Methods:**

This is a retrospective review of surgically treated paediatric meningiomas from three UK centres: the University Hospital of Wales, Alder Hey Children’s Hospital and Royal Manchester Children’s Hospital. Twenty-seven patients aged 16 and under at the time of their first procedure were identified over a 15-year period (1 January 2007 and 1 March 2023). Electronic medical records were used to collect data on age at presentation, sex, location of tumour(s), extent of resection, histology, WHO grade, complications, outcomes and associated conditions, notably neurofibromatosis type 2 (NF2).

**Results:**

Twenty-seven patients underwent 39 procedures. There were 13 males and 14 females. The median age was 13 years (range, 8 months to 16 years). Twenty-one (75%) were WHO grade 1, 6 (21%) were grade 2 and 1 (4%) was grade 3. Eight patients (30%) had confirmed NF2. Twelve patients (44%) were sporadic cases. Twenty-five percent and 50% were the recurrence rate in WHO grade 1 and 2 tumours, respectively.

**Conclusion:**

The risk of grade 1 tumour recurrence was higher than within the adult population. This may be due to histological features of paediatric meningiomas differing from the adult population, and therefore, the WHO grading system may not be reflective of recurrence risk. Future molecular profiling and larger studies are required given the rarity of these cases.

## Introduction

Meningiomas are the most common primary CNS tumour in the adult population, accounting for 39% of reported cases [21]. Ninety-seven to 99% are considered benign (WHO grade 1 and 2) [19, 21, 32]. Within the paediatric population, however, meningiomas are extremely rare, accounting for only 2% of childhood CNS tumours and 1% of all reported meningiomas [14]. Contrary to the adult population, they are known to occur more commonly in males [4, 6, 15]. Studies also suggest that the biological behaviour of meningiomas is more aggressive in up to 25% of cases leading to poorer paediatric patient outcomes in comparison to the adult population [30]. Paediatric meningiomas are associated with neurofibromatosis 2 (NF2) [15, 22, 25] representing over 20% of cases encountered. They are also noted in association with germline mutations of the *NF1*, *BAP1*, *SUFU* and *SMARCE1* genes [28].

Meningiomas are the most common brain neoplasm that is known to be caused by ionising radiation [1, 16], and these tend to occur in younger patients when compared to sporadic adult cases [9], particularly after therapy for medulloblastoma or ependymoma [28]. Radiation-induced paediatric meningiomas account for 8% of all cases. The remaining sporadic cases are rare and less defined within the literature.

Due to the rarity of cases and resultant paucity in the literature, we aimed to retrospectively review the associated conditions, histology, procedures and outcomes of recorded paediatric meningiomas from three neurosurgical units in the UK. We compared our results to relevant paediatric literature with the view to enhance the understanding of paediatric meningiomas within the UK and guide future management.

## Methods

Paediatric patients with diagnosed meningioma/meningiomas were prospectively recorded on databases compiled by each contributing unit. Patients who were aged 16 and under at presentation and had undergone surgical management were identified. Patients who moved out of the area and lost to follow-up were excluded.

Electronic medical records were retrospectively reviewed. Data collected included age of presentation, sex, location of tumour(s), date of surgery/surgeries, extent of resection, histological typing, WHO grade (as per latest WHO guidelines at time of diagnosis), use of radiotherapy at any time point during follow up, date of recorded tumour recurrence, other conditions (notably NF), complications and date of death.

Tumour location was categorised as either skull base, non-skull base (convexity) or spinal. Extent of resection was reviewed case by case. Simpson grading was not consistently recorded; therefore, extent of resection was recorded as complete resection (encompassing Simpson grade 1/2), near total resection (encompassing Simpson grade 3 / > 90% resection) or partial resection (encompassing Simpson grade 4). Tumour recurrence was recorded if there was noted radiological tumour growth which required further management within the follow-up period of this study. Associated and other conditions, i.e. NF2, were diagnosed as per the relevant specialist team at the respective unit providing the patient data. All complications were recorded which ranged from transient neurological deficit to severe and permanent neurological deficits.

### Statistical analysis

Statistical analysis was undertaken using GraphPad Prism Software version 10.1.1 (270). Fishers exact test was used for cohort comparison within the data. Multivariate logistic regression was used when multiple variables were analysed for significance, i.e. tumour grade and extent of resection.

## Results

### Demographics

Twenty-seven patients who initially presented aged 16 and under with surgically managed meningiomas were identified at the University Hospital of Wales, Alder Hey Children’s Hospital and Royal Manchester Children’s Hospital over a 15-year period (1 January 2007 and 1 March 2023), having undergone a total of 39 procedures. There were 13 males (48%) and 14 females (52%). The median age at the time of the first procedure was 13 years (range, 8 months to 16 years and 2 months). Twenty-nine meningiomas in 27 patients were recorded. Two patients had both a skull base and a non-skull base tumour. Two patients had meningiomatosis and only the location of the lesion which was resected or biopsied was described. Three (10%) tumours were spinal, 11 (38%) were in the skull base and the remaining 15 (52%) tumours were non-skull base.

Table 1 summarises the findings of the 27 paediatric meningioma patients from their primary surgery. Two patients (12 and 13) were managed with biopsy and proton beam therapy and did not undergo any further surgical treatment. Of the 27 tumours that underwent surgical resection, 18 (67%) had their tumour completely resected, 5 (18%) achieved near-total resection and 4 (15%) had partial removal/debulking surgery.

**Table 1:** Summary table of 27 paediatric meningiomas

Patient number	Year of surgery	Age at Surgery (years)		Sex	Location	Extent of 1st resection	WHO grade	Tumour histology	Associated genetic conditions or previous radiotherapy	Recurrence (Y/N)	Number of procedures	Adjuvant Therapy (Y/N)
1	2019		11	M	Spinal	Complete	1	Transitional	NF2	Y	2	N
2	2014		11	F	Spinal	Debulk	1	Transitional	NF2	Y	2	N
3	2009		10	M	Spinal	Debulk	1	−	NF2	N	1	N
4	2013		12	M	Non-skull base	Complete	1	−	NF2	N	1	N
5	2022		5	F	Non-skull base	Complete	1	−	NF2	N	1	N
6	2013		13	M	Skull base	Debulk	1	Transitional	NF2	N	1	N
7	2021		13	M	Skull base	Complete	1	Transitional	NF2	Y	2	N
8	2018		12	M	Skull base	Near total	1	Meningothelial	Complex NF2	N	1	N
9	2012		14	F	Non-skull base	Complete	1	Meningothelial	Likely Mosaic NF2^a^	N	1	N
10	2013		14	F	Skull base	Near total	2	Clear cell	SMARCE1 + Schwannomatosis	Y	2	N
11	2013		1	M	Non-skull base	Complete	2	Rhabdoid	Meningiomatosis	N	1	N
12	2019		3	M	Non-skull base	Biopsy	-	−	Meningiomatosis	N	1	Proton beam therapy primary treatment
13	2014		12	F	Skull base	Biopsy	1	Transitional	NF2-like tumour predisposition syndrome-negative for recognised mutations	N	1	Proton beam therapy primary treatment
14	2019		15	M	Non-skull base	Complete	1	Fibrous	Previous radiotherapy for ependymoma	N	1	N
15	2020		15	M	1 Skull base, 1 non-skull base	Near total	1 + 2	Grade 2 = atypical	Previous surgery, radiotherapy, and chemotherapy for medulloblastoma	N (both)	2	N
16	2014		13	F	1 Skull base, 1 non-skull base	Complete / Near total	1 + 2	Meningothelial + Atypical		Y (both)	4	Y
17	2008		13	F	Non-skull base	Complete	2	Chordoid		Y	2	N
18	2013		14	F	Non-skull base	Complete	1	−		N	1	N
19	2016		16	F	Non-skull base	Complete	1	Meningothelial		N	1	N
20	2008		0.7	M	Non-skull base	Complete	1	−		N	1	N
21	2023		15	F	Non-skull base	Complete	1	Meningothelial		N	1	N
22	2015		6	F	Non-skull base	Complete	2	Atypical		N	1	N
23	2021		11	M	Non-skull base	Debulk	1	Transitional		N	1	N
24	2007		15	F	Skull base	Complete	1	−		Y	3	Y
25	2015		11	F	Skull base	Complete	3	Rhabdoid		Y	2	N
26	2022		15	F	Skull base	Complete	1	Meningothelial		N	1	N
27	2021		11	M	Skull base	Complete	1	Transitional		N	1	N

^a^Likely mosaic based on the modified Baser criteria [3]

### Tumour histology

**Fig. 1:**
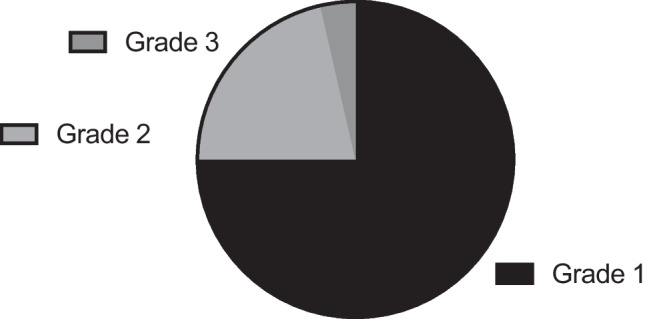
Graph to demonstrate meningioma by WHO grade (*n* = 28)

**Fig. 2:**
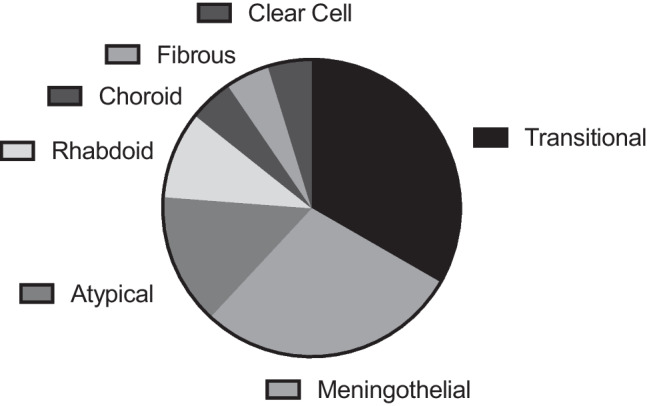
Graph to demonstrate meningioma by histological subtype (*n* = 21)

Tumours were graded according to the WHO classification of tumours of the central nervous system being used at that time point, one sample was not graded. Twenty-one (75%) were WHO grade 1, 6 (21%) were WHO grade 2 and 1 tumour (4%) was WHO grade 3 (Fig. 1). Twenty-one tumours underwent further histological analysis to reveal tumour cell type. The most common cell type was transitional cell (33%), followed by meningothelial (29%), atypical (14%), rhabdoid (9%), chordoid (5%), clear cell (5%) and fibrous (5%) (Fig. 2).

There were 9 cases (33%) of the 27 surgically resected meningiomas which required redo resection. One patient having recurrence of two separate meningiomas. Five of the 20 (25%) resected WHO grade 1 meningioma patients experienced a recurrence, comparatively 3 of the 6 patients (50%) with grade 2 tumours experienced a recurrence. There was only 1 patient with a grade 3 meningioma, who did experience tumour recurrence. Comparison of grade 1 and ≥ grade 2 tumours are demonstrated in Table 2. On comparing these cohorts, there was no statistical significance in comparing patient sex, tumour location, ability to achieve complete resection and complications. Notably, tumour recurrence was not statistically more likely in higher grade meningiomas in this series (*p* = 0.1751). There was also no significance when considering WHO grade and extent of resection (debulking and near total) on multivariate logistic regression analysis (*p* = 0.1042, 0.7415 and 0.4860, respectively). On comparing skull base and non-skull base tumours (*n* = 24), there was no significance found for achieved rates of complete resection (*p* = 0.3926), recurrence (*p* = 0.085) and complications (*p* = 0.4279).

**Table 2:** Comparison of grade 1 and ≥ grade 2 tumour cohorts

Variables	Grade 1	> Grade 2	*p*-value
Age (median)	13	13	
Female sex	9/20 (45%)	5/7 (71.4%)	0.3845
Location;			0.565
Spinal	3/20 (15%)	0/7 (0%)	
Skull base	8/20 (40%)	2/7 (28.6%)	
Non skull base	9/20 (45%)	5/7 (71.4%)	
Complete resection	14/20 (70%)	4/7 (57.1%)	0.6527
Recurrence	5/20 (25%)	4/7 (57.1%)	0.1751
Complications	7/20 (35%)	4/7 (57.1%)	0.3913

*N* = 27 excluding biopsy only patients. Factors compared are median age, proportion of female sex, tumour locations, achievement of complete resection, tumour recurrence and complications

### Associated genetic conditions

Patients received genetic diagnosis at their respective units. Eight patients (30%) had confirmed NF2 recorded, 1 patient was classified as ‘likely NF2 mosaicism’ based upon the Baser criteria [3] and another tested negative for recognised NF2 mutations but there was high clinical suspicion for an NF2-like tumour predisposition disorder. One patient had a confirmed SMARCE1 variant known to cause an inherited disorder of multiple clear cell meningiomas [27]. Two patients had meningiomatosis, and two patients previously underwent radiotherapy of the cranial vault for a medulloblastoma and an ependymoma. Twelve patients were therefore classified as sporadic. On comparing sporadic and non-sporadic cohorts (Table 3), there were more females in the sporadic group, although this was not significant (*p* = 0.0542*)*. There was also no statistical significance comparing tumour location (*p* = 0.3681), ability to achieve complete resection (*p* =  > 0.999), recurrence rate (*p* = 0.646) or complication rate (*p* = 0.4657).

**Table 3:** Comparison of non-sporadic and sporadic cohorts

Variables	Non-Sporadic	Sporadic	*p*-value
Age (median)	13	12.5	
Female sex	5/15 (33.3%)	9/12 (75%)	0.0542
Location;			0.3681
Spinal	3/16 (18.9%)	0/13 (0%)	
skull base	6/16 (37.5%)	5/13 (38.5%)	
Non skull base	7/16 (43.8%)	8/13 (61.5%)	
Complete resection	7/14 (50%)	11/13 (84.6%)	> 0.999
Recurrence	4/14 (28.6%)	5/13 (38.5%)	0.6946
Complications	5/16 (31.3%)	6/13 (46.2%)	0.4657

Factors compared are median age (*n* = 27), proportion of female sex (*n* = 27), tumour locations (*n* = 29), achievement of complete resection (*n* = 27), recurrence (*n* = 27) and complications (*n* = 29). Biopsy only cases were excluded from the analysis of resection and recurrence

### Complications

Of the 39 procedures that the 27 patients underwent, 2 patients received post-operative adjuvant radiotherapy. Eleven (28%) procedures had complications. One patient developed seizures, 3 patients had mild neurological deficits (all improved on follow up), 1 patient had a superficial wound infection and 4 patients required temporary/permanent CSF diversion due to wound leak. One patient required halo fixation following spinal destabilisation, and there was 1 reported infarct with neurological deficit. As noted above, there was no statistical significance in complication rate between sporadic and non-sporadic cohorts, grade 1 and ≥ grade 2 tumours or skull base and non-skull base tumours.

## Discussion

The results from this study are in keeping with current literature. Kotecha et al.’s meta-analysis is the largest of paediatric meningiomas to date. This meta-analysis of paediatric meningiomas from 1989 to 2010 revealed a male to female ratio of 1.3:1 [15]; our study demonstrated a slight female preponderance, though this was minimal (ratio 0.9:1). This remains very different to the strong female preponderance seen in the adult population. However, on analysing subgroups of our cohort, there were higher percentages of females in the sporadic and higher-grade tumour groups. The median age of presentation of the case series was 13 years, with the majority of cases presenting in adolescence; this is in keeping with the current literature [8, 20, 31]. One infantile meningioma was observed in this case series which is a similar to the incidence seen in previous studies [15, 23, 29].

The prevalence of NF2 was 30%, which is more than double the meta-analysis’ figure of 14.5% [15] and higher than other literature reviews [28]. This can be accounted for when considering that Manchester Children’s Hospital is the national paediatric NF2 centre which cares for NF2 patients across the UK. Manchester cohort provided 75% of the NF2 patients. Despite having the largest cohort of patients (*n* = 15), confirmed NF2 prevalence was higher in its sub cohort (40%) in comparison to Cardiff (16.7%) and Liverpool (16.7%). NF2 patients are reportedly more likely to experience a relapse and tend to have lower overall survival [15]. One third of patients with recurrence in our series had NF2; however, this was not significant compared to those who had recurrence without an NF2 diagnosis. Sixty-three percent of confirmed NF2 patients in this study did not have recurrence.

There were no cases of NF1 documented in this case series. Multiple case series have documented a prevalence of meningiomas in paediatric patients with NF1 [10, 29], including 3.4% of patients in the previously mentioned meta-analysis. There is no strong evidence for an association between NF1 and meningioma; however, the incidence of NF1 in meningioma patients from the meta-analysis is much higher than the background incidence at 1 in 2600 to 3000 [7]. Within our study, the absence of NF1 patients is likely due to the rarity of cases in general. Within Europe, there are established guidelines for cranial and spinal imaging in NF1 patients, possibly accounting for high diagnostic pick up rate. All our centres follow and contribute to these guidelines, and therefore, prevalence should be similar [5].

Kotecha et al. categorise tumour location into supratentorial, infratentorial and spinal, which to compare with our study has been approximated into non-skull base, skull base and spinal, respectively. Both studies place non-skull base as the most common location, followed by skull base and spinal. Proportions are similar: 52%, 38%, 10% in our study vs 83.1%, 11.3%, 5.6% in the meta-analysis [15]. There is a higher proportion of skull base and spinal meningiomas in our study likely due to the increased proportion of NF2 patients. Paediatric patients with NF2 presenting with new meningiomas have a higher skull base prevalence of 30.9%, contrasting to a 19.1% skull base prevalence in adults with NF2 [17]. All spinal meningiomas in this study were associated with NF2, but there was no significance noted in tumour locations between our sporadic and non-sporadic group.

Revision to the WHO grading system in 2016 and 2021 has made comparison of tumours by grade over time difficult due to meningiomas being placed in different categories based on parameters which vary with invasion, histological features and cell typing, e.g. all brain-invasive meningiomas are now classified as grade 2, along with chordoid and clear-cell types [18]. WHO grading from this case series was taken from the time of grading so are in accordance with the latest WHO grading available at the time of the assessment. 78.9% of patients in Kotecha et al.’s meta-analysis had WHO grade 1 meningiomas [15]. Our case series demonstrated similar results with 75% having WHO grade 1 histology. However, 21% of patients in our case series had WHO grade 2 and 4% had WHO grade 3 meningiomas; in comparison to Kotecha et al.’s, 9.9% were grade 2 and 8.9% were grade 3. We suspect that the difference between grade 2 and 3 meningiomas compared to the meta-analysis is due to the subjectivity in the grading of meningiomas prior to the revision of WHO grading in 2016.

In this case series, WHO grading was not effective in predicting the likelihood of tumour recurrence nor was extent of tumour resection. Twenty-five percent of grade 1 and 50% of grade 2 tumours experienced recurrence and required further resection within the study follow up time frame. There was only 1 patient with grade 3 histology who also experienced recurrence. Kotecha et al. experienced a tumour recurrence in 24% of WHO grade 1 tumours, 6% of WHO grade 2 tumours and 18% of grade 3 tumours (mean follow-up 5.7 years) [15]. The higher grade 2 recurrence rate in our study, using primarily the 2016 grading system, may demonstrate the improved prognosticative capabilities of the revised WHO grading systems. However, there may be limitations within the paediatric population given the higher rates of grade 1 recurrence when compared to the adult population. Haddad et al. undertook a retrospective review of grade 1 meningiomas in adults between 2007 and 2017; their recurrence rate was 10.9%, nearly half of what is noted within our and Kotecha et al.’s paediatric cohorts [11]. Although their follow-up period within their study is shorter, if Haddad et al. had used the 2016 classification, this grade 1 recurrence rate would likely be lower as more would be classified as grade 2.

Histological cell type is becoming less important in predicting tumour behaviour and its management. The most common subtype of meningioma in our series was transitional, followed by meningothelial. The meta-analysis agrees with this; however, the proportions differ: 33% and 29% in our series and 21.1% and 20.7% in the meta-analysis. Fourteen percent of meningiomas in our series had an atypical histological cell type compared to only 6.8% of those in the meta-analysis. There is an association of NF2 with an increased number of atypical and anaplastic type meningiomas [2, 22]. Despite having a high proportion of NF2 patients in our cohort, this association was not seen. Two out of 3 atypical meningiomas were sporadic, and 1 was associated with previous radiotherapy. Over half of the higher-grade tumours in this series were sporadic. There was no clear association between more aggressive histological type and NF2 diagnosis in our series. There was no clear association between histological subtype and recurrence, although numbers are too small in our series to have significance.

Huntoon et al. looked at radiological features of sporadic paediatric meningioma, noting atypical features which were more likely to denote higher grade. The transitional cell type remained the most common in both the typical and atypical feature group [12]. Conversely, Kirches et al. who looked specifically at the molecular profile of paediatric tumours identified a much higher proportion of aggressive subtypes, with 57% of the 37 cases having an atypical histological subtype, concluding that paediatric histology is generally more aggressive than an adult cohort [13].

On genetic analysis, Kirches et al. noted that there were frequent NF2 alterations. Alterations of the tumour suppressor gene NF2, located at 22q12.2, are commonly encountered in adult meningiomas; however, there is increased frequency noted within paediatric meningiomas [28]. Other genetic alterations, exclusive of NF2 that are reported in sporadic adult meningiomas, include TRAF7, SMO, KLF4, AKT1 and PIK3CA. In their study, they did not detect any alteration on these genes from paediatric samples, suggesting that these are exclusive to the pathogenesis of adult meningiomas. They further reported no TERT promoter mutations, a marker of aggressive meningioma in adults. Interestingly, they noted three novel subgroups of meningioma based on DNA methylation profiles. The first group contained largely clear cell variants, the second group was predominantly atypical WHO grade 2 meningiomas in which all samples had alterations on chromosome 22 and there was the highest frequency of patients diagnosed clinically with NF2. The final group, 2b, was mixed but contained all high grade rhabdoid meningiomas. These groups are distinct from the 6 subgroups described in adult meningiomas, establishing that paediatric tumours are dissimilar from adults in both subtype and molecular profiling [13]. They reported no YAP1 genetic alteration within their results, however. YAP1 is an oncogene located at 11q22.1 chromosome. Alterations to YAP1 have been reported in 9 paediatric meningiomas, specifically sporadic cases lacking in NF2 alterations [26]. Meningioma subtype was variable, and no further classifications could be drawn, highlighting the need for further in-depth analysis of paediatric tumours to guide diagnostics and future therapies.

As of March 2023, all patients in the case series were alive. In Kotecha et al.’s meta-analysis, death occurred in 84 of the 664 patients with recorded outcomes (12.7%) [15]. This case series’ reduced mortality rate could be contributed to advances in surgical monitoring, peri-operative care and adjuvant treatments over the last decade. There is little documentation of the complications in the literature as complications leading to death are the only complications discussed in the meta-analysis [15]. Grossbach et al. [10], Fan et al. [8] and Santos et al. [24] state that no surgical complications were observed in their paediatric series; however, they have not defined these parameters. Our complication rate was 28% which covered mild anticipated complications such as temporary neurological deficit to rarer more serious adverse events such as infarct and spinal instability. There was no significance in complication rates between tumour grade, location or sporadic vs. non-sporadic cases.

## Limitations

Due to the rarity of paediatric meningiomas, the sample size is small despite the involvement of three centres over a long timeframe. Data gathered in this case series was intended to add to current literature and provide a larger sample for future systematic reviews and meta-analyses. A longer study time would be required to obtain an accurate picture of tumour recurrence. Longer follow-up will always provide improved accuracies for tumour recurrence data, but particularly with slower growing tumours in a population where the natural history is not defined This study is also limited by incomplete data sets due to a lack of further testing, i.e. histological analysis. The Royal Manchester Children’s Hospital is also the national centre for paediatric NF2 patients, which may disproportionately affect the documented number of NF2 patients in comparison to the UK population.

## Conclusion

This study presented a series of paediatric meningiomas. The case series concurs with most of the current paediatric literature; however, mortality seems to have improved over time and NF2 patients had less recurrence. These findings may be explained due to improved surgical techniques, superior imaging modalities, widespread use of neuro-navigation and improved post-operative care.

We noted that the risk of grade 1 tumour recurrence was higher than within the adult population likely because histological features of paediatric meningioma differ from the adult population. Therefore, the WHO grading system may not be reflective of tumour risk, particularly recurrence. This will become more apparent as more molecular profiling is undertaken on future cases. Further multicentre studies are required for improved understanding and management of meningiomas in paediatric populations.

## Data Availability

No datasets were generated or analysed during the current study.
